# Intermittent fasting inhibits platelet activation and thrombosis through the intestinal metabolite indole-3-propionate

**DOI:** 10.1093/lifemeta/loaf002

**Published:** 2025-01-29

**Authors:** Zhiyong Qi, Luning Zhou, Shimo Dai, Peng Zhang, Haoxuan Zhong, Wenxuan Zhou, Xin Zhao, Huajie Xu, Gang Zhao, Hongyi Wu, Junbo Ge

**Affiliations:** Department of Cardiology, Zhongshan Hospital, Fudan University, Shanghai Institute of Cardiovascular Diseases, 180 Fenglin Road, Shanghai 200032, China; National Clinical Research Center for Interventional Medicine, 180 Fenglin Road, Shanghai 200032, China; Department of Cardiology, Zhongshan Hospital, Fudan University, Shanghai Institute of Cardiovascular Diseases, 180 Fenglin Road, Shanghai 200032, China; National Clinical Research Center for Interventional Medicine, 180 Fenglin Road, Shanghai 200032, China; Department of Cardiology, Zhongshan Hospital, Fudan University, Shanghai Institute of Cardiovascular Diseases, 180 Fenglin Road, Shanghai 200032, China; National Clinical Research Center for Interventional Medicine, 180 Fenglin Road, Shanghai 200032, China; Department of Cardiology, Zhongshan Hospital, Fudan University, Shanghai Institute of Cardiovascular Diseases, 180 Fenglin Road, Shanghai 200032, China; National Clinical Research Center for Interventional Medicine, 180 Fenglin Road, Shanghai 200032, China; Department of Cardiology, Zhongshan Hospital, Fudan University, Shanghai Institute of Cardiovascular Diseases, 180 Fenglin Road, Shanghai 200032, China; National Clinical Research Center for Interventional Medicine, 180 Fenglin Road, Shanghai 200032, China; Department of Cardiology, Zhongshan Hospital, Fudan University, Shanghai Institute of Cardiovascular Diseases, 180 Fenglin Road, Shanghai 200032, China; National Clinical Research Center for Interventional Medicine, 180 Fenglin Road, Shanghai 200032, China; Department of Cardiology, Zhongshan Hospital, Fudan University, Shanghai Institute of Cardiovascular Diseases, 180 Fenglin Road, Shanghai 200032, China; National Clinical Research Center for Interventional Medicine, 180 Fenglin Road, Shanghai 200032, China; Department of Infectious Disease, Zhongshan Hospital, Fudan University, 180 Fenglin Road , Shanghai 200032, China; Department of Cardiology, Zhongshan Hospital, Fudan University, Shanghai Institute of Cardiovascular Diseases, 180 Fenglin Road, Shanghai 200032, China; National Clinical Research Center for Interventional Medicine, 180 Fenglin Road, Shanghai 200032, China; Department of Cardiology, Zhongshan Hospital, Fudan University, Shanghai Institute of Cardiovascular Diseases, 180 Fenglin Road, Shanghai 200032, China; National Clinical Research Center for Interventional Medicine, 180 Fenglin Road, Shanghai 200032, China; Department of Cardiology, Zhongshan Hospital, Fudan University, Shanghai Institute of Cardiovascular Diseases, 180 Fenglin Road, Shanghai 200032, China; National Clinical Research Center for Interventional Medicine, 180 Fenglin Road, Shanghai 200032, China; Institutes of Biomedical Sciences, Fudan University, 131 Dong’an Road, Shanghai 200032, China; Key Laboratory of Viral Heart Diseases, National Health Commission, 180 Fenglin Road, Shanghai 200032, China

**Keywords:** intermittent fasting, indole-3-propionate, platelet activation, arterial thrombosis, PXR

## Abstract

Platelet hyperreactivity contributes significantly to thrombosis in acute myocardial infarction and stroke. While antiplatelet drugs are used, residual ischemic risk remains. Intermittent fasting (IF), a dietary pattern characterized by alternating periods of eating and fasting, has shown cardiovascular benefits, but its effect on platelet activation is unclear. This study demonstrates that IF inhibits platelet activation and thrombosis in both patients with coronary artery disease and apolipoprotein E (*ApoE*) knockout (*ApoE*^*−/−*^) mice, by enhancing intestinal flora production of indole-3-propionic acid (IPA). Mechanistically, elevated IPA in plasma directly attenuates platelet activation by binding to the platelet pregnane X receptor (PXR) and suppressing downstream signaling pathways, including Src/Lyn/Syk and LAT/PLCγ/PKC/Ca^2+^. Importantly, IF alleviates myocardial and cerebral ischemia/reperfusion injury in *ApoE*^*−/−*^ mice. These findings suggest that IF mitigates platelet activation and thrombosis risk in coronary atherosclerosis by enhancing intestinal flora production of IPA, which subsequently activates the platelet PXR-related signaling pathways.

## Introduction

Arterial thrombosis, a critical step in diseases like acute myocardial infarction (MI) and stroke, involves platelet activation, accumulation at injury sites, and subsequent clot formation [1, 2]. While risk factors, such as atherosclerosis, hyperlipidemia, and hyperglycemia, contribute to increased platelet reactivity and thrombosis risk [3–5], the exact mechanisms behind this hyperactivation remain elusive. This lack of understanding of platelet hyperactivation is particularly concerning given the rise in morbidity from coronary artery disease (CAD), despite declining mortality rates [6]. Although antiplatelet therapy has been widely used, patients continue to suffer from acute MI, where platelet activation and resulting arterial blood clot formation are the common pathological processes [7]. Further research is needed to explore the involvement of other inhibitory mechanisms in regulating platelet activation during MI.

Intermittent fasting (IF), a popular dietary pattern involving cyclic periods of eating and fasting, has gained significant interest for its potential health benefits. The common strategies for IF include 5:2 IF (60% energy restriction on two days per week) and 1:1 IF (60% energy restriction every other day). Numerous studies have demonstrated that IF has benefits for a variety of diseases, including diabetes [8, 9], hyperlipidemia [10], cancer [11], Alzheimer’s disease [12], as well as aging [13, 14]. Emerging evidence suggests that IF may influence various physiological processes affecting the cardiovascular system [15], such as blood pressure reduction [16–18], decrease in circulating cholesterol and triglycerides [18–21], moderation of insulin resistance [8, 16], and increase in heart rate variability [22]. However, the specific effects of IF on platelet activation and thrombosis risk remain relatively unexplored. One potential mechanism by which IF might exert its effects lies in the modulation of gut microbiota and its metabolites. Prior studies have shown that IF alters the composition of gut bacteria and the metabolites they produce, potentially impacting disease progression [23–28]. Based on these findings, we investigated whether these IF-induced alterations in gut metabolites could regulate platelet activation and thrombosis.

This study investigated the role of IF in regulating platelet reactivity and thrombosis, as well as the underlying mechanisms. We demonstrated that the IF diet significantly inhibited platelet activation and thrombus formation. Mechanism studies revealed a significant increase in indole-3-propionic acid (IPA) levels in IF mice using metabolomics. We then explored the effects of IPA on platelet activation and found that IPA activated platelet pregnane X receptor (PXR) and downregulated the PXR-mediated Src/Lyn/Syk (Src tyrosine kinase/Lck/Yes-related novel protein tyrosine kinase/spleen tyrosine kinase) and LAT/PLCγ/PKC/Ca^2+^ (linker for activation of T cells/phospholipase Cγ/protein kinase K/Ca^2+^) signaling pathways, ultimately inhibiting platelet activation and thrombosis. Notably, the IF diet alleviated microvascular obstruction and lessened myocardial damage post-ischemia/reperfusion (I/R) injury in apolipoprotein E (*ApoE*) knockout (*ApoE*^*−/−*^) mice. These findings suggest that IF is a potential dietary therapy for patients with coronary atherosclerosis by inhibiting platelet activation and thrombosis via the gut microbiota-derived metabolite IPA.

## Results

### IF inhibits platelet activation and thrombosis in CAD patients and *ApoE*^*−/−*^ mice

**Figure 1:**
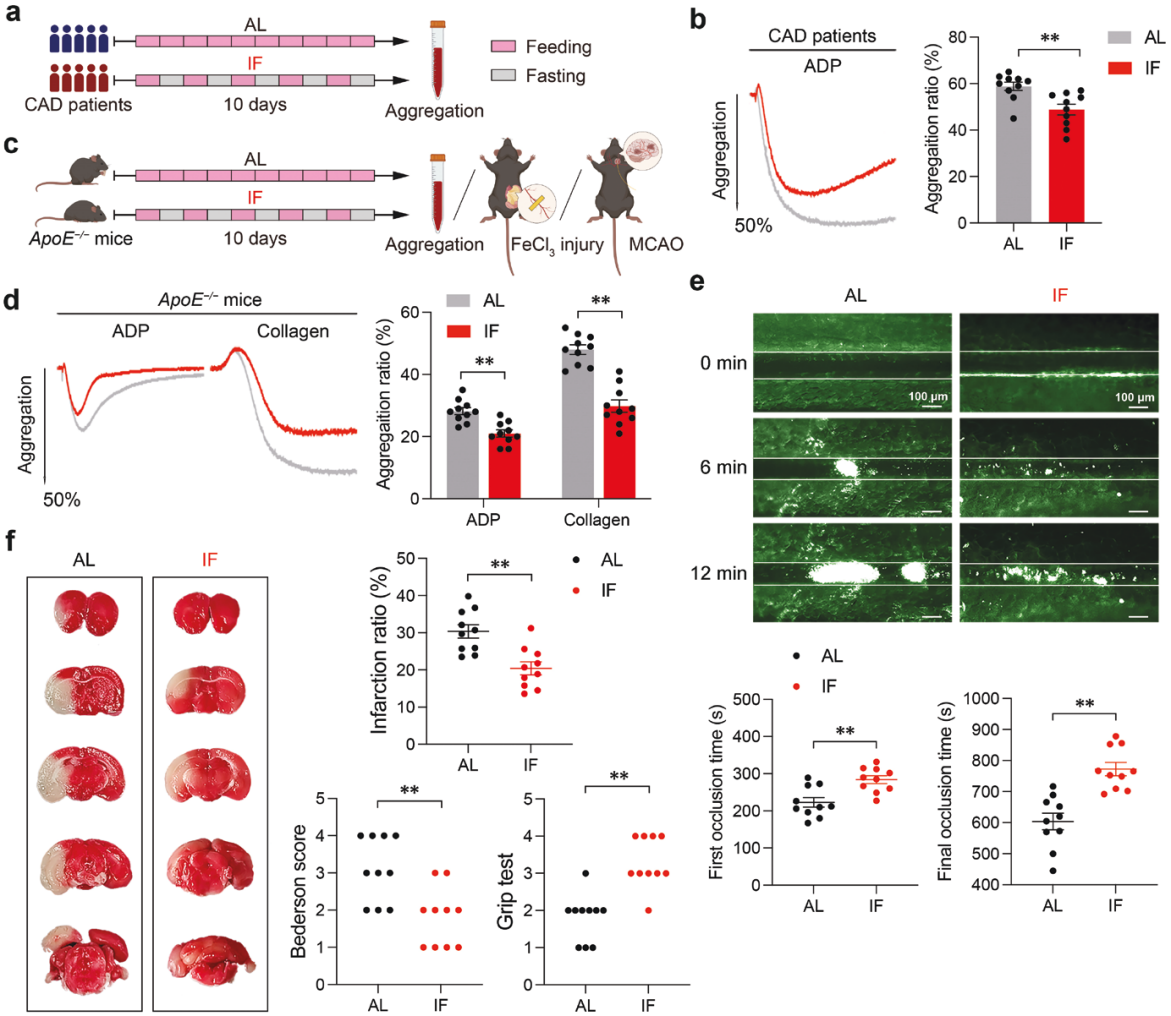
IF attenuates platelet activation and thrombosis *in vivo*. (a and b) Schematic diagram of AL or IF treatment for CAD patients (a) and representative platelet aggregation tracings of PRP induced by 10 μmol/L ADP (b). CAD patients underwent 10 consecutive days of AL or IF diet and their blood was drawn for platelet preparation and aggregation (*n* = 10, biologically independent individuals per group). (c) Schematic diagram of experiments presented in (d), (e), and (f). *ApoE*^*−/−*^ mice were randomly treated with AL or IF diet for 10 days and then used for platelet aggregation (d), FeCl_3_-injured thrombus formation in mesenteric arteriole (e) experiments, or MCAO models (f). (d) Representative tracings and summary results of platelet aggregation in AL and IF-diet *ApoE*^*−/−*^ mouse induced by 10 μmol/L ADP or 0.5 μg/mL collagen, using PRP (*n* = 10, biologically independent animals per group). (e) Representative images of FeCl_3_-injured thrombus formation in AL and IF-diet *ApoE*^*−/−*^ mice at 0, 6, and 12 min, respectively. Summary results of the first and final occlusion time are presented below (*n* = 10, biologically independent animals per group). (f) Representative pictures of the MCAO model conducted on AL and IF-diet *ApoE*^*−/−*^ mice. Summary results of infarction ratio, Bederson score, and Grip test performed 24 h after MCAO are shown (*n* = 10, biologically independent animals per group). Data are shown as mean ± SEM. ^**^P < 0.01. Data were analyzed using unpaired Student’s *t*-test (b, d, e, and infarction ratio in f) and *U* test (Bederson score and Grip test in f).

To investigate the impact of IF on platelet activation in the context of atherosclerosis, aggregation ratios of platelets from CAD patients were tested before and after 10 days of IF or *ad libitum* (AL) diet (Fig. 1a). Notably, IF significantly inhibited aggregation induced by ADP and collagen of platelets from CAD patients (Fig. 1b). *ApoE*^*−/−*^ mice have been widely used to as animal models of atherosclerosis and hyperlipidemia. The same phenomena were also observed in *ApoE*^*−/−*^ mice treated with AL or IF diet (Fig. 1c and d). Consistently, *ApoE*^*−/−*^ mice undergoing 10-day IF exhibited decreased thrombus formation when their mesenteric arterioles were injured by FeCl_3_, in comparison with AL-diet mice (Fig. 1e). To examine the impact of IF on infarct size and neurological function in cerebral ischemia, we utilized the middle cerebral artery occlusion (MCAO) model. IF-diet *ApoE*^*−/−*^ mice exhibited significantly fewer infarct volumes, lower neurological deficit scores, and better performance on the rotarod test compared to the controls (Fig. 1f). To rule out the effect of acute fasting on platelet function, we collected platelets from CAD patients and *ApoE*^*−/−*^ mice with an IF or AL diet during the fed day of the IF cycle. As expected, we also observed significant inhibition of platelet activation in IF groups (Supplementary Fig. S1). To avoid the influence of total caloric intake, we calculated the food consumption and found that there was little difference between the two groups (data not shown). Taken together, these results indicated that IF diet alleviates platelet activation and arterial thrombus formation *in vivo*, driving us to detect the underlying mechanism.

### Metabolites vary in mice fed with the IF and AL diets

**Figure 2:**
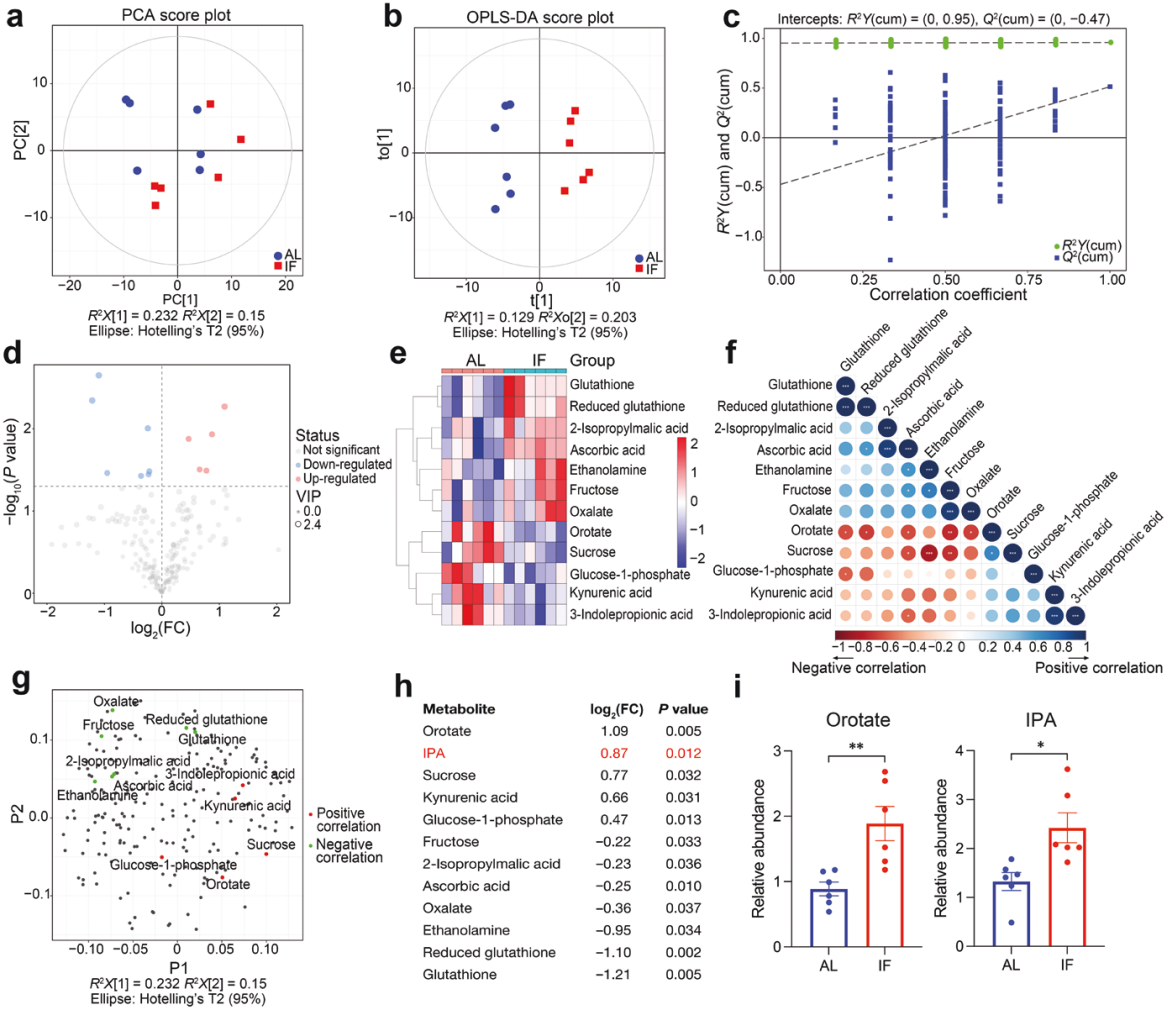
Plasma metabolites in IF-diet mice differ from those in AL-diet mice according to untargeted metabolomics analysis (UTMs). (a and b) Score scatter plots of PCA model (a) and OPLS-DA model (b) for IF versus AL group. These two models indicate the levels of clustering and dispersion of the two groups. (c) The permutation test result of OPLS-DA model for the two groups. The values of *R*^2^*Y* (approximately to 1.0) and *Q*^2^ (over 0.5) represent the satisfactory predictability and interpretability of the model, respectively. (d) The volcano plot illustrates the differentially abundant metabolites between the two groups. (e) The hierarchical cluster analysis heatmap of differentially abundant metabolites between the two groups. (f) The correlation heatmap of the correlation between differentially abundant metabolites. The point size reflects the Pearson coefficient. (g) The PCA loading plot showing the primarily differential metabolites in the plasma of IF group compared to AL group. The overlap between loadings was modeled using variable correlation analysis. (h) The table lists differentially abundant metabolites in (g). The logarithmic fold changes (log_2_(FC)) and *P* values of *t*-test are presented (*n* = 6, biologically independent animals per group). (i) Summary results of relative abundance of orotate and IPA, the most differentially abundant metabolites between the IF and AL groups (*n* = 6; unpaired *t*-test; ^*^P < 0.05; ^**^P < 0.01).

Given that the IF diet potentially contributes to alterations of gut microbiota and the metabolites [23–28], we identified metabolites potentially responsible for IF-induced inhibition of platelet activation, by performing liquid chromatography–mass spectrometry (LC–MS) metabolomic analysis on serum samples from mice fed with either an IF or an AL diet. Principal component analysis (PCA) and orthogonal projections to latent structures-discriminant analysis (OPLS-DA) revealed distinct clustering patterns between the two groups (Fig. 2a and b). The OPLS-DA model demonstrated good predictability and interpretability with an *R*^2^ of 0.958 and a *Q*^2^ of 0.514 (Fig. 2c). As shown in the volcano plot (Fig. 2d), the hierarchical cluster analysis heatmap (Fig. 2e), the correlation heatmap (Fig. 2f), and the PCA loading plots (Fig. 2g), among the 335 metabolites detected, 12 metabolites were differently abundant between the groups, including five upregulated metabolites and seven downregulated ones. Prominently, the two most abundant metabolites in the serum of IF-diet mice were orotate (*P* = 0.005) and IPA (*P* = 0.012; Fig. 2h and i). Further results showed that orotate had no direct effect on platelet activation at different doses (Supplementary Fig. S2). We, therefore, focused on detecting the IPA levels in the plasma and platelets from CAD patients undergoing AL and IF diets by LC–MS. The results showed that IF treatment significantly increased the plasma and intracellular IPA levels (Supplementary Fig. S3). Therefore, IPA, a gut-derived metabolite, was considered next to have the potential to attenuate platelet activation and thrombosis during IF.

### IPA directly inhibits platelet activation *in vitro*

**Figure 3:**
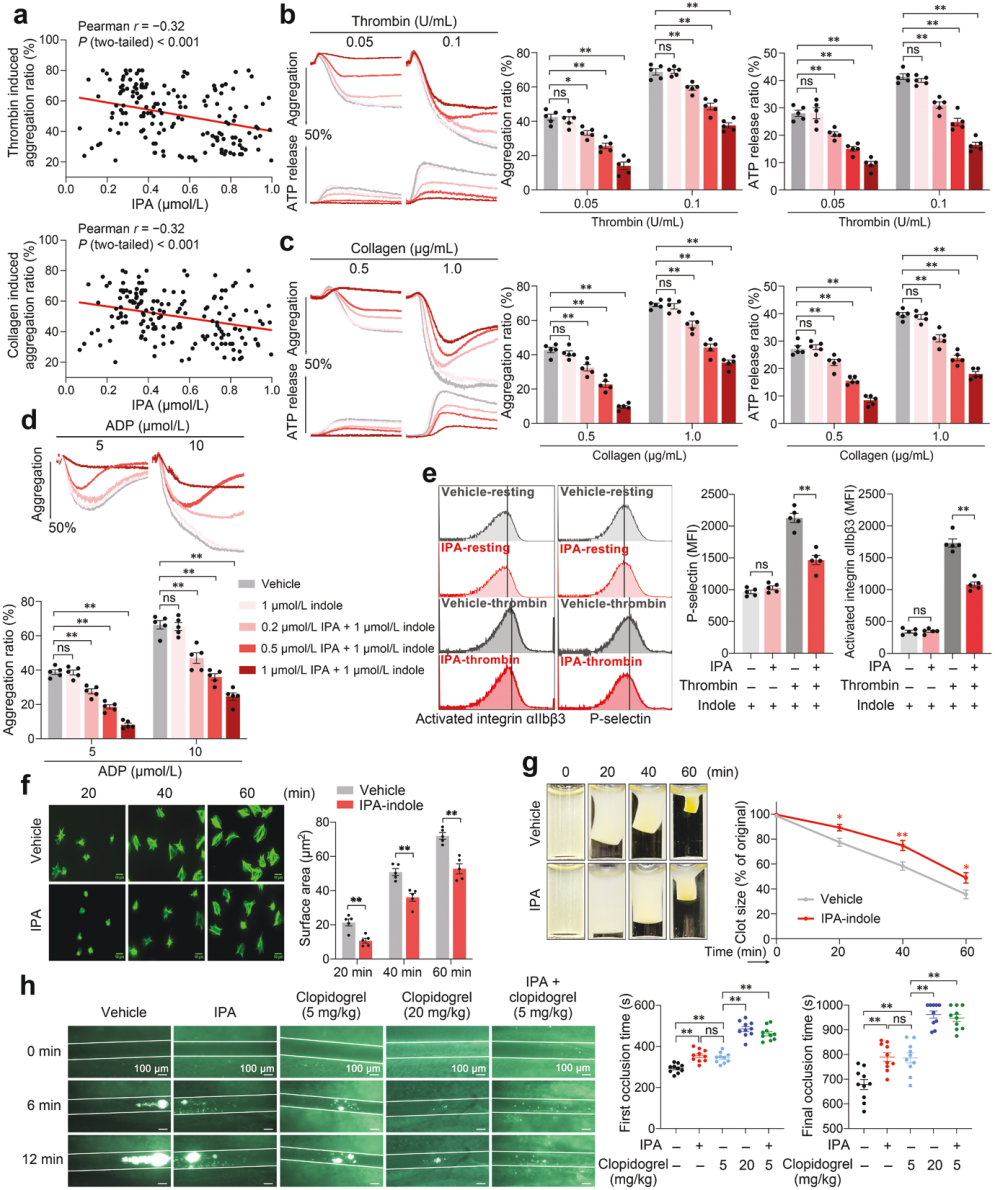
IPA directly attenuates platelet activation and thrombus formation both *in vitro* and *in vivo*. (a) Correlation analysis plots of IPA levels and platelet aggregation ratios using linear regression and Pearson correlation model. Platelet aggregation in PRP from 160 patients with CAD was induced by thrombin (0.2 U/mL) or collagen (1.0 μg/mL). IPA concentrations were tested by UPLC-MS. Each point represents a single individual. (b−d) Aggregation of platelets treated with or without IPA induced by agonists. Washed human platelets (300 × 10^9^/L) incubated with vehicle, 1 μmol/L indole, or 1 μmol/L indole with 0.2, 0.5, or 1 μmol/L IPA for 20 min were stimulated by thrombin (0.05 or 0.1 U/mL) (b), collagen (0.5 or 1.0 μg/mL) (c), or ADP (5 or 10 μmol/L) (d). Simultaneously, platelet dense granule secretion induced by thrombin or collagen was quantified by ATP release labeled with luciferin. Representative tracings and summary results are presented (*n* = 5, biological replicates). (e) Activation of platelet integrin αⅡbβ3 and P-selectin release from platelet α-granules induced by thrombin. Washed human platelets (10 × 10^9^/L) were incubated with vehicle or 0.5 μmol/L IPA (in the presence of 1 μmol/L indole) and then stimulated with 0.05 U/mL thrombin. Representative histogram and summary results were obtained using flow cytometry for PAC-1 binding and CD62P expression (*n* = 5, biological replicates). (f) Platelet spreading with or without IPA. Washed human platelets (20 × 10^9^/L) were spread on immobilized fibrinogen and stained with FITC-labeled phalloidin, and 0.5 μmol/L IPA and 1 μmol/L indole were used. Representative pictures and summary results of spreading areas (μm^2^) are presented (*n* = 5, biological replicates). (g) Clot retraction with or without IPA. Washed human platelets (500 × 10^9^/L) were stimulated with thrombin in the presence of human PPP and Ca^2+^. IPA (0.5 μmol/L) and 1 μmol/L indole was used. Representative photographs and summary results of clot area percentage at different time points are presented (*n* = 5, biological replicates). (h) Intravital microscopy of FeCl_3_-injured thrombosis in WT mouse mesenteric arteriole with or without IPA or clopidogrel. WT mice randomly receiving vehicle, IPA (0.2 μg per mouse, i.v., single dose), clopidogrel (5 or 20 mg/kg, p.o., single dose), or clopidogrel (5 mg/kg) plus IPA (0.2 μg per mouse) were used for injection of calcein-labeled platelets and FeCl_3_ injury 4 h later. Typical thrombus formation at 6 and 12 min after FeCl_3_ injury is shown. The final and first (time to the first thrombus formation > 20 μm and stable for > 2 min) occlusion time was analyzed (*n* = 10, biologically independent animals per group). Data are shown as mean ± SEM. *ns*, no significance; ^*^P < 0.05; ^**^P < 0.01. Data were analyzed using simple linear regression in the correlation analysis (a), two-way ANOVA followed by Tukey’s multiple comparisons test (b, c, d, f, and g), and one-way ANOVA followed by Tukey’s multiple comparisons tests (e and h).

To further investigate the function of IPA in platelet activation, we included 160 patients with a clinical diagnosis of CAD who were not taking antiplatelet medications for at least 14 days before blood collection and found that their plasma IPA levels were negatively correlated with their platelet aggregation ratios (Fig. 3a). Baseline characteristics of the analyzed patients are summarized in Supplementary Table S1.

To test the effect of IPA on washed human platelet activation and simulated internal conditions, indole was used to combine with IPA and stimulate human PXR (hPXR), the receptor of IPA in platelets [29], as IPA alone can only weakly activate hPXR [30]. Of note, no significant differences were observed in plasma indole levels between IF and AL groups in CAD patients and *ApoE*^*−/−*^ mice (Supplementary Fig. S4). In the presence of 1 μmol/L indole, physiologically achievable low (0.2 μmol/L), middle (0.5 μmol/L), and high (1 μmol/L) concentrations of IPA [31] attenuated platelet aggregation induced by ADP, thrombin, or collagen, and ATP release from dense granule induced by thrombin or collagen in a dose-dependent manner. However, 1 μmol/L indole alone did not affect platelet activation induced by agonists (Fig. 3b−d). As indicated, two concentrations of each agonist were used for confirmation in the above platelet aggregation experiments.

Upon vascular injury, platelets encounter agonists that trigger their respective receptors to initiate signal transduction pathways. Despite their distinct initiation points, these pathways converge into a unified response, culminating in the activation of integrin αⅡbβ3 and granule secretion via inside-out signaling. Subsequently, ligand binding to the activated integrin initiates outside-in signaling, driving platelet spreading, clot retraction, and further granule release [32]. By analyzing procaspase-activating compound 1 (PAC-1) binding and P-selectin (CD62P) expression, we found that, in the presence of indole, middle concentration of IPA (0.5 μmol/L) inhibited both activation of integrin αⅡbβ3 and P-selectin release from α-granules in washed human platelets stimulated by thrombin (Fig. 3e). As the early phase of integrin outside-in signaling [33], human platelet spreading on fibrinogen was weakened by middle concentration of IPA (0.5 μmol/L) with indole (Fig. 3f). The integrin-mediated spreading is followed by a later outside-in signaling event during platelet activation, which involves Talin-1 interaction with the integrin β3 intracellular domain and is associated with clot retraction. Consistently, IPA attenuated human platelet clot retraction (Fig. 3g). These evidences support that IPA directly inhibits human platelet activation *in vitro*.

### IPA attenuates thrombus formation *in vivo*

Having established IPA’s ability to inhibit platelet activation, we investigated its potential impact on thrombus formation *in vivo*. Wild-type (WT) mice received intravenous injections of IPA to elevate plasma IPA levels. Subsequently, FeCl_3_-induced thrombus formation in mesenteric arterioles was assessed at various time points. As shown in Fig. 3h, IPA treatment significantly prolonged the time to the first thrombus formation and final occlusion. To compare its effectiveness as an antithrombotic drug, clopidogrel was used as a positive control. Both 5 mg/kg and 20 mg/kg clopidogrel doses significantly extended the time to the first thrombus formation and final occlusion in a dose-dependent manner. Strikingly, IPA displayed antithrombotic efficacy comparable to 5 mg/kg clopidogrel. More importantly, the combination of IPA and clopidogrel demonstrated a superior antithrombotic effect compared to either treatment alone. IPA and clopidogrel showed a potential synergistic effect in terms of antithrombotic efficacy, suggesting that IPA may be potent for inhibiting thrombosis.

### IPA interacts with PXR in platelets to inhibit platelet activation

**Figure 4:**
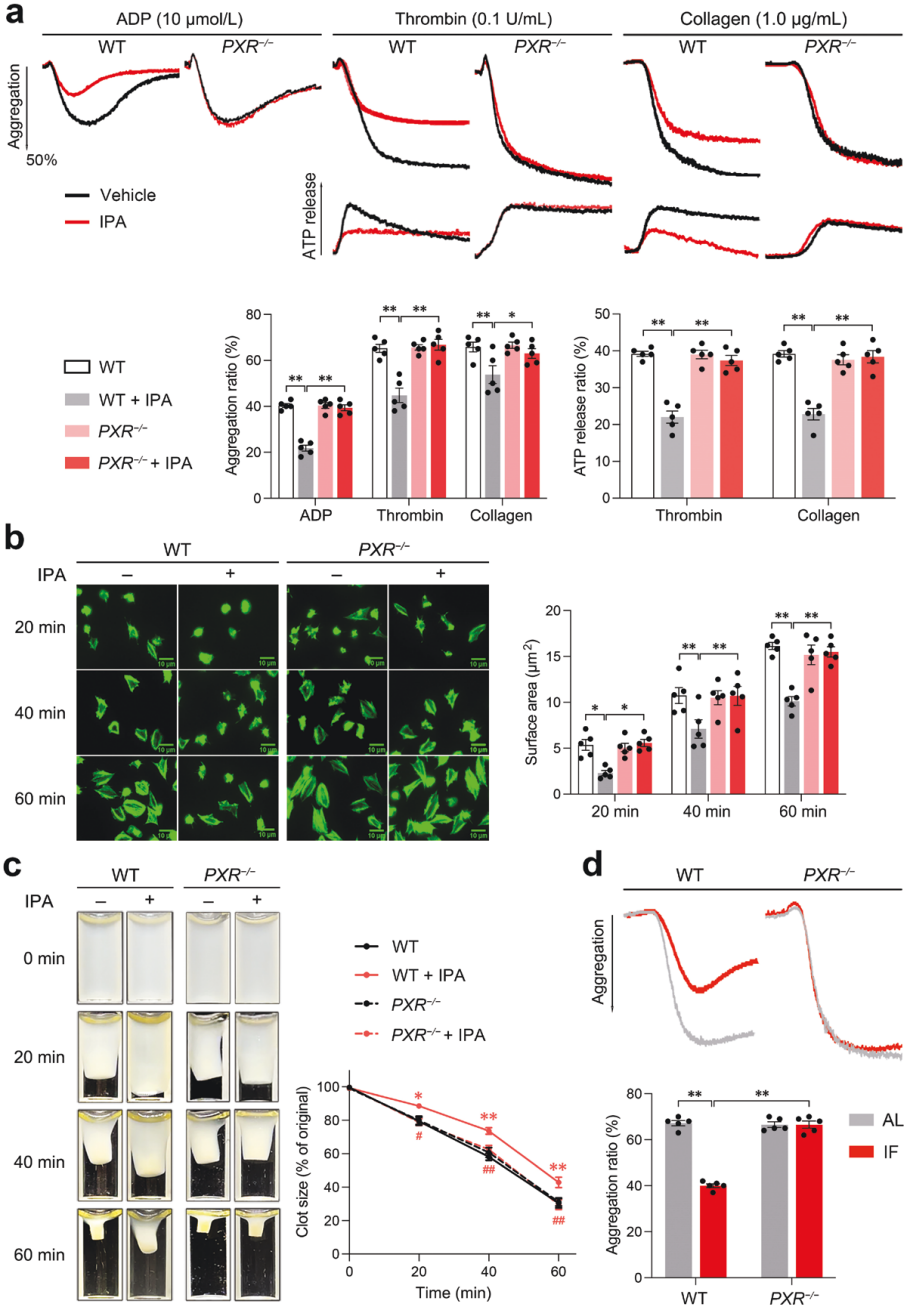
IPA inhibits platelet activation by binding to PXR in platelets. (a) Platelet aggregation and ATP release of WT and *PXR*^*−/−*^ mice with or without IPA. Washed platelets from WT or *PXR*^*−/−*^ mice were incubated with IPA or vehicle for 20 min and then stimulated with ADP (10 μmol/L), thrombin (0.1 U/mL), or collagen (1.0 μg/mL). IPA (0.5 μmol/L) was used. Representative platelet aggregation, ATP release tracings, and summary data are presented (*n* = 5, per group). (b) Platelet spreading on fibrinogen of WT and *PXR*^*−/−*^ mice with or without IPA. IPA (0.5 μmol/L) was used. Respective pictures and the summary results of the spreading area at different time points are presented (*n* = 5, per group). (c) Clot retraction of WT and *PXR*^*−/−*^ mice with or without IPA. IPA (0.5 μmol/L) was used. Representative photographs and summary data of clot area percentage at different time points are presented (*n* = 5, per group; The *P* values are represented by ^*^/^**^ for WT versus WT + IPA, and ^**#**^/^**##**^ for WT + IPA versus *PXR*^−*/*−^ + IPA). (d) Platelet aggregation of WT and *PXR*^*−/−*^ mice treated with AL or IF diet induced by collagen. After a 10-day AL or IF diet (see schematic diagram in Fig. 1c), the mouse PRP was prepared for aggregation experiment. Representative platelet aggregation tracings and summary data are presented (*n* = 5, per group). Data are shown as mean ± SEM. ^*(**#**)^*P* < 0.05; ^**(**##**)^*P* < 0.01. Data were analyzed using two-way ANOVA followed by Tukey’s multiple comparisons test (a, b, c, and d).

Our initial findings demonstrate that IPA inhibits platelet activation and attenuates thrombosis *in vivo*. To understand the mechanisms behind this, we investigated how IPA might suppress platelet activation. IPA is known to directly interact with the PXR in enterocytes [30]. PXR is a nuclear receptor in platelets and has a potential to negatively regulate platelet functions [29]. We hypothesized that IPA inhibits platelet activation by binding to platelet PXR and activating downstream signaling pathways. To test this, we investigated whether PXR is required for the dampening effects of IPA on platelet activation. Since IPA is a strong agonist of mouse PXR (mPXR) [30], we incubated mouse platelets with IPA without indole. In agreement with the results obtained with washed human platelets, IPA significantly attenuated mouse platelet aggregation and ATP release stimulated by ADP, thrombin, or collagen (Fig. 4a). Importantly, these potentiating effects were significantly diminished in *PXR*-deficient (*PXR*^*−/−*^) platelets, suggesting that IPA inhibits platelet activation in a PXR-dependent mechanism. Similarly, the inhibitory effects of IPA on platelet spreading (Fig. 4b) and clot retraction (Fig. 4c) were also dependent on PXR. These findings suggest that IPA may bind to PXR in platelets, thereby inhibiting platelet activation. Furthermore, consistent with our expectations, *PXR*^*−/−*^ mice subjected to IF did not exhibit any alterations in platelet aggregation (Fig. 4d), implying that IF attenuates platelet activation by increased production of IPA acting on platelet PXR. Furthermore, we found that plasma IPA levels after 10-day IF treatment in WT and *PXR*^*−/−*^ mice were significantly higher than those before the treatment (Supplementary Fig. S5).

**Figure 5:**
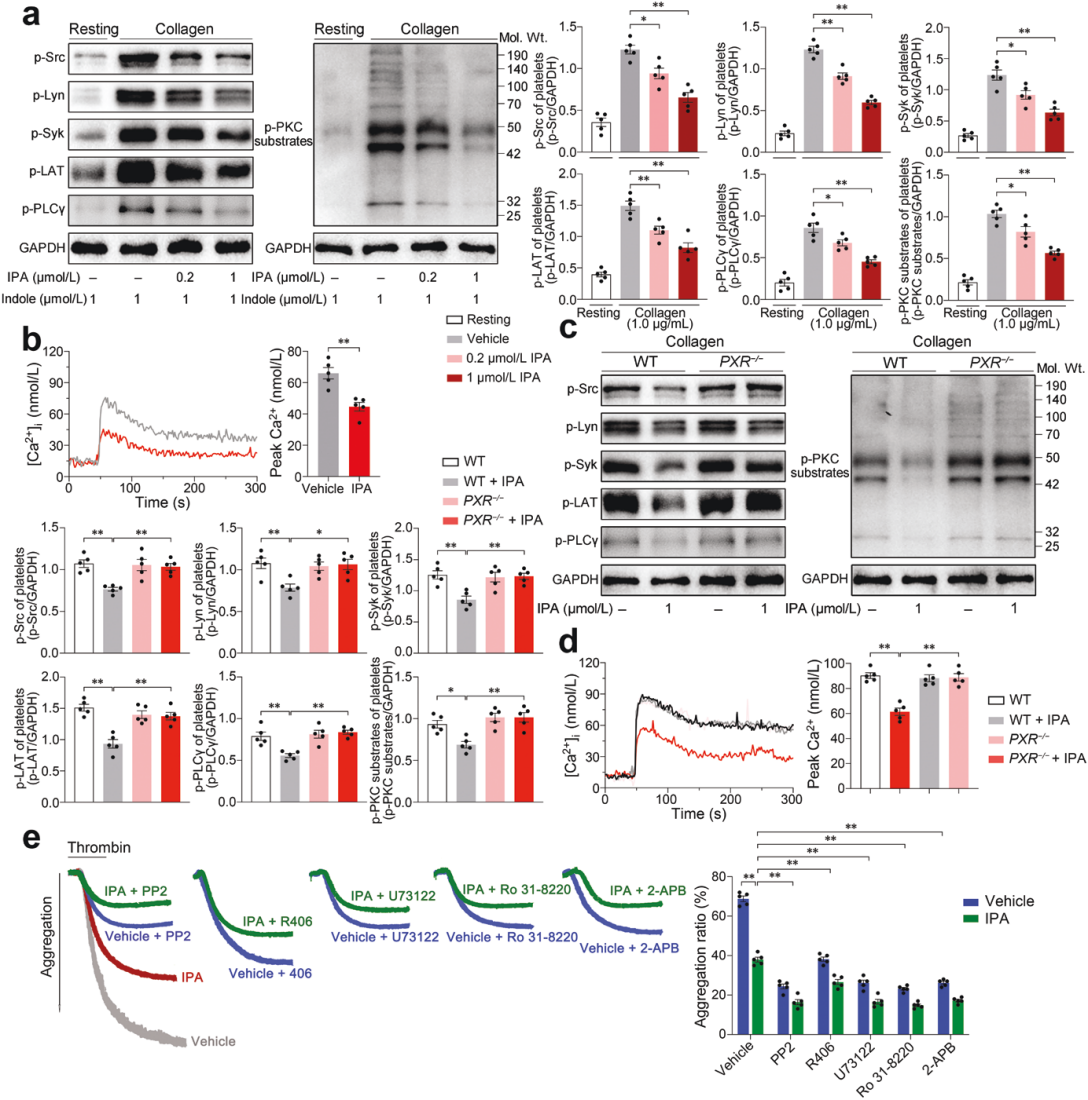
IPA attenuates downstream signaling pathways of platelet PXR, including the decreased phosphorylation of Src, Lyn, Syk, LAT, PLCγ, and PKC, as well as the reduced Ca^2+^ influx. (a) Western blot analysis of phosphorylated Src, Lyn, Syk, LAT, PLCγ, and PKC, as well as GAPDH in activated human platelets incubated with different concentrations of IPA (in the presence of indole) and resting platelets. After incubation with IPA or vehicle, washed human platelets were stimulated by collagen (1.0 μg/mL) and then prepared for western blotting analysis. IPA (0.2 μmol/L and 1 μmol/L) and indole (1 μmol/L) were used. Representative results and summary data are presented (*n* = 5, biologically independent individuals per group). (b) Ca^2+^ influx of human platelets incubated with or without IPA induced by thrombin. Washed human platelets loaded with Fura-2 were incubated with IPA or vehicle for 20 min and then measured for 300 s with thrombin (0.1 U/mL). IPA (0.5 μmol/L) and indole (1 μmol/L) were used. Representative results and summary data are presented (*n* = 5, biologically independent individuals per group). (c) Western blot analysis of phosphorylated Src (Y418), Lyn (Y397), Syk (Y525/526), LAT (Y200), PLCγ (Y1217), and PKC, as well as GAPDH in activated platelets from WT and *PXR*^*−/−*^ mice incubated with or without IPA. Representative results and summary data are presented (*n* = 5, biologically independent animals per group). (d) Ca^2+^ influx of platelets from WT and *PXR*^*−/−*^ mice incubated with or without IPA induced by thrombin. Representative results and summary data are presented (*n* = 5, biologically independent animals per group). (e) Platelet aggregation with IPA or inhibitors targeting signaling pathways downstream PXR in platelets. Washed human platelets were pretreated with 10 μmol/L PP2 (Src inhibitor), 10 μmol/L R406 (Syk inhibitor), 10 μmol/L U73122 (PLCγ inhibitor), 10 μmol/L Ro 31-8220 (PKC inhibitor), and 50 μmol/L 2-APB (Ca^2+^ inhibitor) for 10 min, then treated with 1 μmol/L IPA (with 1 μmol/L indole) or vehicle as control before stimulation with 0.1 U/mL thrombin. Representative aggregation tracings and summary data are presented (*n* = 5, biologically independent individuals per group). Data are shown as mean ± SEM. ^*^P < 0.05; ^**^P < 0.01. Data were analyzed using one-way ANOVA followed by Tukey’s multiple comparisons test (a, c, and d), unpaired Student’s *t*-test (b), and two-way ANOVA followed by Tukey’s multiple comparisons test (e).

To elucidate the mechanisms underlying IPA’s inhibition of platelet activation, we investigated the effects of IPA on PXR-related downstream signaling. Platelet activation is a complex process involving multiple signaling pathways. Ligand binding to PXR in platelets inhibits the phosphorylation of Src family kinases (SFKs) (Src at Y418 and Lyn at Y397; downstream of integrin αⅡbβ3 and α2β1, C-type lectin-like receptor 2 (CLEC-2), Fc gamma receptor ⅡA (FcRγⅡA), and glycoprotein Ⅰb-Ⅸ-Ⅴ (GPⅠb-Ⅸ-Ⅴ) receptor) and the downstream signaling pathway of glycoprotein Ⅵ (GPⅥ, receptor of collagen), leading to subsequent reduction of phosphorylated Syk at Y525/526, LAT at Y200, PLCγ at Y1217 and PKC, as well as the attenuation of calcium metabolism, and integrin outside-in signaling ultimately [29]. Therefore, collagen was used as the platelet agonist for the following mechanism analysis. IPA concentration-dependently attenuated the phosphorylation of Src (Y418), Lyn (Y397), Syk (Y525/526), LAT (Y200), PLCγ (Y1217), and PKC in washed human platelets stimulated by collagen (Fig. 5a) and Ca^2+^ influx stimulated by thrombin (Fig. 5b). IPA similarly attenuated the phosphorylation of signaling molecules in mouse platelets stimulated by collagen, and Ca^2+^ influx stimulated by thrombin. However, these effects were significantly alleviated in *PXR*^*−/−*^ mice (Fig. 5c and d).

To determine whether PXR-related signaling pathways contribute to the effects of IPA on platelet activation, we blocked Src, Syk, PLCγ, PKC, and calcium channels with their specific inhibitors. A significant increase in IPA-induced platelet aggregation was observed after the inhibitors were introduced (Fig. 5e), suggesting a synergistic effect between IPA and inhibitors of PXR-related signaling pathways. Collectively, these results support that PXR-related signaling pathways in platelets contribute to the suppressive effects of IPA on platelet activation.

### *Clostridium sporogenes* recolonization inhibits platelet activation and thrombosis

**Figure 6:**
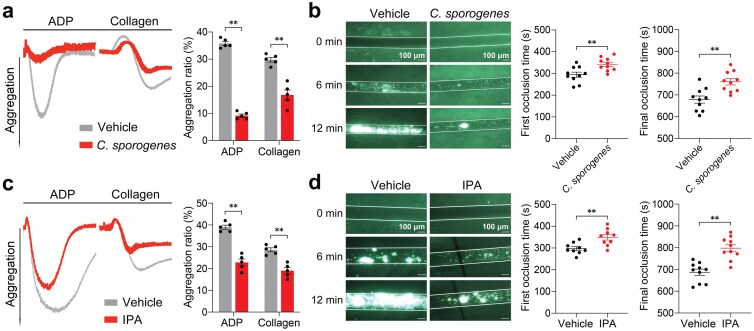
Gavage of *C. sporogenes* and IPA treatment inhibit platelet aggregation and thrombus formation in WT mice. (a and b) Platelet aggregation and FeCl_3_-injured thrombosis in mesenteric arteriole of mice treated with *C. sporogenes* or vehicle by gavage. WT mice were randomly administered with *C. sporogenes* or vehicle (100 μL LB containing 20% glycerol per mouse) for 10 consecutive days and then used for platelet aggregation induced by ADP (10 μmol/L) or collagen (0.5 μg/mL) in PRP and FeCl_3_-injured thrombosis experiments. Representative aggregation tracings and summary data are presented in (a) (*n* = 5, biologically independent animals per group). Typical thrombus formation and the final and first occlusion time are presented in (b) (*n* = 10, biologically independent animals per group). (c and d) Platelet aggregation and FeCl_3_-injured thrombosis in mesenteric arteriole of mice gavaged with IPA or vehicle. WT mice were randomly treated with IPA (20 mg/kg/day) or vehicle (sterile PBS, 100 μL/day) by gavage for 10 consecutive days, and then used for platelet aggregation in PRP (c) (*n* = 5, biologically independent animals per group) and FeCl_3_-injured thrombosis in mesenteric arteriole (d) (*n* = 10, biologically independent animals per group). Data are shown as mean ± SEM. ^**^P < 0.01. Data were analyzed using unpaired Student’s *t*-test (a, b, c, and d).

Given that IPA is a gut metabolite and has been identified to attenuate platelet activation and thrombosis, we next aimed to detect the impact of gut microbiota alteration on platelet activation and thrombosis. Physiological IPA is primarily produced by the intestinal gram-positive bacterium *C. sporogenes* in mice [34]. Thus, we administrated *C. sporogenes* or vehicle by oral gavage and measured platelet aggregation and thrombus formation. Compared with the control mice, the mice treated with *C. sporogenes* had higher IPA levels in their guts, plasma, and platelets (Supplementary Fig. S6). Consequently, the mice treated with *C. sporogenes* (Fig. 6a and b), along with mice directly administered IPA orally (Fig. 6c and d), displayed significantly lower platelet aggregation ratio and prolonged thrombosis time compared to the vehicle group.

To further verify the causal relationship between IF and alterations in gut microbiota, we detected the intestinal abundance of *C. sporogenes* in AL and IF diet-feeding mice. As expected, *C. sporogenes* increased in the guts of IF diet-feeding mice (Supplementary Fig. S7). Antibiotic treatment significantly eliminated the anti-platelet aggregation effects of IF (Supplementary Fig. S8). These results indicate that IPA derived from *C. sporogenes* may mediate the beneficial effects of IF through inhibiting platelet activation and thrombus formation.

### IF alleviates myocardial I/R injury

**Figure 7:**
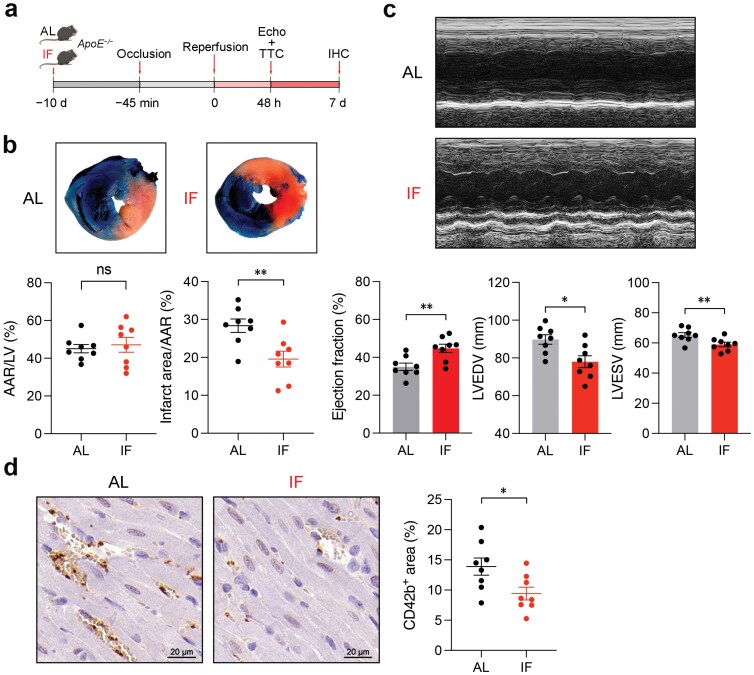
IF alleviates myocardial I/R injury of *ApoE*^*−/−*^ mice. (a) Schematic diagram for experiments in (b), (c), and (d). (b) IF markedly decreases the infarct area/AAR ratio in *ApoE*^*−/−*^ mice. The LV tissue sections of AL and IF-diet mice were stained with TTC and Evans blue dye after I/R injury. The blue area, stained by Evans blue, indicates the non-infarct area, while the other unstained area indicates AAR. The red area, which can be stained by TTC, indicates viable myocardium, and the white area indicates the infarct area. Representative sections and summary data of AAR/LV and infarction area/AAR are presented (*n* = 10, biologically independent animals per group). (c) IF improves cardiac function as indicated by echocardiography, including EF and LV volume indices (LVEDV and LVESV) in *ApoE*^*−/−*^ mice. Representative M-mode echocardiograms and summary data are presented (*n* = 10, biologically independent animals per group). Abbreviations: LVEDV, left ventricular volumes at end diastole; LVESV, left ventricular volumes at end systole. (d) Myocardial I/R-induced microvascular thrombosis in the reperfused cardiac tissue of *ApoE*^*−/−*^ mice is reduced by IF. Representative immunohistochemistry results and summary data of CD42b positive area quantification (as a percentage of the field) are presented (*n* = 10, biologically independent animals per group). Data are shown as mean ± SEM. *ns*, no significance; ^*^P < 0.05; ^**^P < 0.01. Data were analyzed using unpaired Student’s *t*-test (b, c, and d).

Reperfusion of ischemic heart tissue can trigger myocardial I/R injury. Platelet activation and infiltration into the tissue contribute to infarct expansion and worsening heart function [35]. Having shown that IF inhibited platelet hyperreactivity and *in vivo* thrombosis, we next evaluated the potential therapeutic effects of IF on treating myocardial I/R injury. We investigated the effects of IF in an I/R injury model using *ApoE*^*−/−*^ mice undergoing IF for 10 days. Severe myocardial ischemia was induced by a 45-min temporary ligation of the left anterior descending (LAD) coronary artery followed by reperfusion, and the severity of myocardial injury was evaluated by 2,3,5-triphenyl tetrazolium chloride (TTC)/Evans blue staining and echocardiography 48 h later, and immunohistochemistry 2 days later (Fig. 7a). IF significantly reduced the infarct area/area at risk (AAR) ratio compared to AL-diet mice (Fig. 7b). In addition, IF improved heart function, including ejection fraction (EF) and left ventricular (LV) volume indices (Fig. 7c). Immunohistochemistry analysis revealed that IF reduced myocardial I/R-induced microthrombi in the mouse hearts (Fig. 7d). These findings suggest that IF may be a potential dietetic strategy to mitigate myocardial I/R injury and improve outcomes of CAD patients.

## Discussion

**Figure 8:**
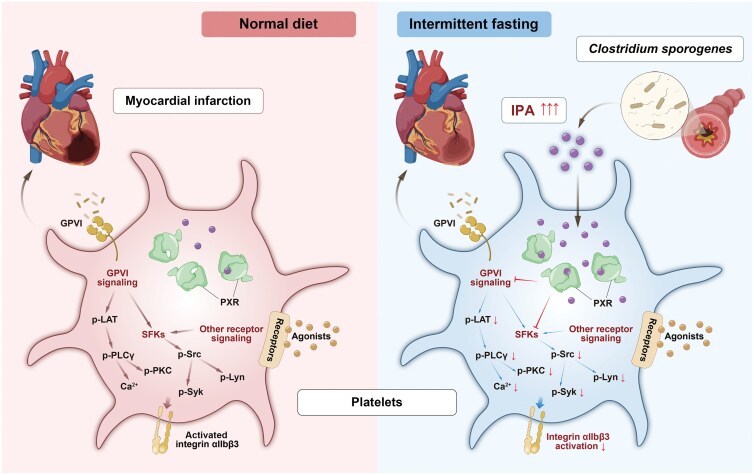
Proposed working model for IF enhancing the production of IPA from intestinal *C. sporogenes*, which directly attenuates platelet activation and thrombosis, as well as alleviates microvascular thrombosis after I/R injury by binding to platelet PXR and activating the downstream signaling. When bodies are undergoing IF, plasma IPA increases significantly, which is primarily produced by gut clostridia. IPA directly inhibits platelet hyperreactivity and thrombosis by binding to platelet PXR and activating PXR downstream signaling, which consequently alleviates microvascular thrombosis during MI. Abbreviations: GPⅥ, glycoprotein Ⅵ; IPA, indole-3 propionate; p-LAT, phosphorylated linker for activation of T cells; p-Lyn, phosphorylated Lck/Yes-related novel protein tyrosine kinase; p-PKC, phosphorylated protein kinase K; p-PLCγ, phosphorylated phospholipase Cγ; p-Src, phosphorylated Src; p-Syk, phosphorylated spleen tyrosine kinase; PXR, pregnane X receptor; SFKs, Src family kinases.

Platelet hyperreactivity is a known risk factor for cardiovascular disease. IF has been detected to have cardiovascular benefits through blood glucose and lipid regulation, blood pressure reduction, and oxidative stress moderation. However, the impact of IF on platelet activation remains elusive. In this study, we found that IF inhibits platelet activation and thrombosis through a gut metabolite IPA-dependent manner. We demonstrated that: (i) IF diet reduces platelet activation and thrombosis in CAD patients and *ApoE*^*−/−*^ mice; (ii) LC-MS metabolomics analysis showed that IF increases mouse serum IPA levels through gut bacterial modulation; (iii) Elevated IPA directly inhibits platelet activation, reduces thrombosis, and contributes to lower platelet activation in CAD patients; (iv) Mechanistically, IPA binds to the platelet PXR, suppressing the PXR-mediated Src/Lyn/Syk and LAT/PLCγ/PKC/Ca^2+^ signaling pathways, ultimately dampening platelet activation; (v) IF diet mitigates mouse microvascular obstruction and myocardial damage following I/R injury (Fig. 8). These findings suggest that IF promotes IPA production, thereby contributing to reduced platelet reactivity and decreased thrombosis and cardiovascular risk.

IF is a dietary pattern gaining significant interest for its potential health benefits. Studies suggest positive impacts on cardiovascular health, including blood pressure reduction [36], dyslipidemia and hyperglycemia regulation [10], weight loss [15], and oxidative stress and inflammation reduction [36, 37]; however, evidence on preventive effects of IF against cardiovascular risk is limited. A randomized controlled trial involving 101 patients with prediabetes and obesity showed significant reductions in body mass index, blood glucose, triglycerides, and low-density lipoprotein cholesterol for both alternate-day fasting and 16/8 time-restricted fasting over 3 weeks [15], suggesting potential benefits for reducing diabetes and cardiovascular disease risk in this population. A meta-analysis by Allaf *et al.*, which included 18 studies with 1125 participants (observation periods ranging from 4 weeks to 6 months), reported modest weight loss benefits of IF compared to unrestricted eating [38]. It is important to note that there are significant uncertainties in the evidence, as well as emerging studies that have contradictory results. A survey of over 20,000 adults, newly released at the 2024 American Heart Association Epidemiology and Prevention Conference, revealed a 91% increased risk of cardiovascular deaths among those who adhered to an 8-h time-restricted eating pattern [39]. Another study found that the early and advanced atherosclerotic lesion formation in *ApoE*^*−/−*^ mice was aggravated after 11 weeks of alternate day fasting due to cholesterol metabolic pathway alteration [40]. Given the conflicting evidence, further research of fully understanding the impact of IF on cardiovascular diseases is needed. In addition, the effects of IF on long-term clinical outcomes, including overall mortality and MI should also be investigated. Our findings indicate that IF-mediated inhibition of platelet reactivity is beneficial for CAD patients, however, clinical intervention is lacking.

A large number of studies have demonstrated that intestinal microbiota composition as well as metabolites derived from the gut are changed under IF dietary pattern, thereby regulating disease progress. Recently, Serger *et al.* identified several gut bacterial metabolites elevated in mice on an IF diet using gas chromatography-mass spectrometry (GC-MS) metabolomic analysis, including 3-indolelactic acid, 2,3-butanediol, xylose, and IPA [41]. Notably, the increased IPA production in IF-diet mice was almost eliminated by vancomycin treatment. These findings strongly support our LC–MS metabolomic data, with orotate and IPA showing the most significant changes in IF mice compared to the controls. IPA has been identified as a tryptophan metabolite produced by gut bacteria. The bacterium *C. sporogenes* plays a key role in IPA production, and its colonization can establish IPA production even in the absence of a complete gut microbiome [42]. Other bacteria like *Peptostreptococcus* and certain *Clostridia* species also possess this conversion ability [43–45]. Studies have shown that platelet activation can be regulated by gut microbial metabolites. Nemet *et al*. found that gut metabolite trimethylamine N-oxide (TMAO) [46] and phenylacetylglutamine (PAGln) enhance platelet hyperreactivity and thrombosis risk [47]. In our study, colonizing mice with *C. sporogenes* resulted in platelet inhibition, consistent with IPA gavage. Current data show that serum IPA levels correlate with intestinal IPA levels [42], suggesting that IPA produced by gut *C. sporogenes* can cross the intestinal barrier and enter the bloodstream, where it exerts its inhibitory effect on platelet activation. These results raise the possibility of using *C. sporogenes* as a therapeutic strategy to increase plasma IPA levels and potentially attenuate platelet function in humans.

PXR is a nuclear receptor expressing in various cell types, including vascular endothelial cells [48], enterocytes [30], and fibroblasts [49]. Recently, PXR was also found in platelets, and several PXR ligands, including SR12813 and rifampicin, have been reported to inhibit platelet reactivity to all kinds of agonists in a non-genomic way [29]. These pathways involve PXR-mediated suppression of SFKs (Src/Lyn/Syk) and downstream signaling cascades of GPⅥ (LAT/PLCγ/PKC/Ca^2+^). However, the precise mechanism of how PXR regulates these signaling pathways is still unclear. IPA has been detected as another ligand of PXR in enterocytes [30] and vascular endothelial cells [48]. Our findings support the hypothesis that IPA attenuates platelet activation by binding to PXR and inhibiting its associated signaling pathways. Notably, research suggests that IPA in combination with indole can significantly activate hPXR, while PXR alone exhibits weak agonistic activity [30]. In washed human platelets, 1 μmol/L indole, a concentration achievable under physiological conditions, is sufficient for IPA to activate PXR. While PXR can function as a nuclear receptor and transcription factor, the long-term effects of IF on PXR-mediated transcriptional regulation in platelets (megakaryocytes) remain unexplored and warrant further investigation.

IPA has emerged as a promising player in CAD management. Recent studies support the protective role of IPA in cardiovascular health. Xue *et al*. demonstrated that IPA deficiency contributes to atherosclerotic cardiovascular disease, potentially by promoting reverse cholesterol transport and hindering atherosclerotic plaque formation [31]. Similarly, a cohort study conducted by Li *et al*. observed a significant association between higher plasma IPA levels and reduced cardiovascular and all-cause mortality in patients with CAD [50]. The anti-inflammatory properties of IPA may also offer additional benefits in CAD. In animal models, IPA is found to effectively ameliorate left heart dysfunction and myocardial inflammation associated with sepsis by modulating the AhR/NF-κB/NLRP3 (aryl hydrocarbon receptor/nuclear factor-kappaB/NOD-like receptor protein 3) signaling pathway [51]. A similar result comes from a peripheral artery disease (PAD) cohort study by Ho *et al*., which identified a negative association between IPA levels and both peripheral atherosclerosis and major adverse cardiac events [52]. Collectively, these studies suggest that IPA’s ability to reduce atherosclerosis holds promise for atherosclerosis management. Our findings further add to this growing body of evidence by demonstrating IPA’s capacity to alleviate platelet hyperreactivity and thrombosis. This highlights the need for future investigations exploring the potential link between IPA levels, CAD prognosis, and platelet reactivity in patients.

In conclusion, this study sheds light on a novel mechanism by which IF benefits cardiovascular health. We found that IF reduces platelet activation and thrombosis by increasing the production of IPA, a gut bacteria-derived metabolite. IPA binds to the platelet PXR, suppressing PXR-mediated signaling pathways (Src/Lyn/Syk and LAT/PLCγ/PKC/Ca^2+^), ultimately dampening platelet activation. This mechanism is consistent with previous studies on the effects of IF on gut microbiota and metabolite production. These findings strongly suggest that on the foundation of antiplatelet medications, IF as a dietary regimen to mitigate platelet activation presents the potential for a wider spectrum of clinical applications deserving of further investigation.

## Limitations of the study

The current evidence for IF as a potential dietary treatment for CAD is still insufficient. In the future, we will conduct clinical intervention studies to explore the feasibility and availability of IF on patients with CAD.

## Materials and methods

### Human participants

All experiments involving human subjects adhered to the Declaration of Helsinki and received approval from the Institutional Review Board of Zhongshan Hospital, Fudan University. Before written consent was obtained, each subject was informed about the study design and possible risks. For the correlation analysis, we recruited 160 patients with a clinical diagnosis of CAD who had not taken antiplatelet medications for at least 14 days. Additional exclusion criteria included: heart failure; active cancer; severe hepatic or renal insufficiency; and hematologic disorders, for example, thrombocytopenia or anemia. The baseline characteristics of the patients are presented in Supplementary Table S1.

### Mice

All animal procedures were conducted in accordance with the National Institutes of Health Guidelines for the Care and Use of Laboratory Animals (NIH Publication No. 85-23, revised 1996) and were approved by the Animal Care and Use Committee of Zhongshan Hospital, Fudan University. WT C57BL/6 mice were purchased from Shanghai Jie Si Jie Laboratory Animals (Shanghai, China). *ApoE*^*−/−*^ mice and *PXR* knockout (*PXR*^*−/−*^) mice on C57BL/6J background were purchased from Cyagen Biosciences (Suzhou, China).

### Reagents

ADP, thrombin, collagen, fibrinogen, and luciferin were from Chrono-Log (Havertown, PA). Human fibrinogen, FITC-labeled phalloidin, calcein acetoxymethyl ester, orotate, and IPA were purchased from Sigma-Aldrich (St Louis, MO). PP2, R406, U73122, Ro 31-8220, and 2-aminoethyl diphenylborinate (2-APB) were from MedChemExpress (New Jersey, USA). The antibodies utilized in the study are listed in Supplementary Material.

### IF in humans and mice

CAD patients with aspirin treatment were randomly assigned to AL or IF treatment groups. The IF group was instructed to fast every second day since randomization and to have an AL diet on the alternating days. Blood samples were collected before and after the 10-day experiment. After blood collection, the platelets were promptly prepared for further experiment [29].

Similarly, *ApoE*^*−/−*^ C57BL/6 mice of 6–8 weeks with matched body weight were randomized to the AL and IF groups. The IF group was fed as described [29]. Briefly, food was withheld every second day during observation. Mouse platelets were prepared right after the last fasting day.

### IPA treatment in mice

To investigate how oral IPA intake affects mouse platelet activation in Fig. 6, the C57BL/6 mice were treated with 20 mg/kg IPA (diluted to 2.5 mg/mL in sterile PBS) or sterile PBS (control) daily by gavage. For intravital microscopic examination of FeCl_3_-injured thrombosis as shown in Fig. 3, mice were treated with 0.2 μg per mouse by intravenous injection.

### *Clostridium sporogenes* recolonization

*Clostridium sporogenes* (ATCC, 15579) were cultured overnight in LB broth at 37°C under anaerobic conditions. The bacterial suspension was prepared in 100 µL LB broth containing 20% glycerol. Mice received 100 µL of this suspension by oral gavage daily for 10 consecutive days. Control mice received 100 µL LB broth containing 20% glycerol only.

Mice were housed in individually ventilated cages under specific pathogen-free conditions with a 12-h light/12-h dark cycle. They were provided with standard rodent chow and water *ad libitum*.

### MCAO model

A previously described method was used to induce cerebral ischemia [53–55]. Briefly, *ApoE*^*−/−*^ mice were anesthetized with an intraperitoneal injection of 1.25% tribromoethanol (0.2 mL/10 g body weight). The right internal, external, and common carotid arteries were carefully ligated, followed by the insertion of a monofilament nylon filament (Guangzhou Jialing Biotechnology Co., Ltd.) into the internal carotid artery bifurcation via the external carotid artery. After 1 h of ischemia, reperfusion was initiated by removing the filament and loosening the suture ligature on the common carotid arteries. Mice with a Zea Longa score of 2 were considered to have successfully undergone the procedure and were included in subsequent experiments. Functional assessments, including the Bederson score and Grip test for neurological and motoric functions, respectively, and infarct volume evaluation were performed 24 h after reperfusion.

### Metabolomics by LC–MS analysis

Mouse peripheral blood was collected in an EDTA tube and promptly transferred to ice before being centrifuged at 12 000 rpm for 20 min at 4°C. The supernatant plasma was collected and stored at −80°C before metabolite analysis was conducted on LC–MS (Thermo, Ultimate 3000LC, Q Exactive) platform.

The procedures of sample preparation, extract analysis, metabolite identification, and quantification were conducted at Sensichip Biotechnology Co., Ltd. (Shanghai, China). The final data were processed by SIMCA-P software (V14.1, Sartorius Stedim Data Analytics AB, Umea, Sweden). To be brief, the PCA and OPLS-DA models were used to explore the differentially abundant metabolites between the AL and IF groups, followed by the OPLS-DA permutation test to determine the predictability and interpretability of the model. *P* values < 0.05 were considered statistically significant.

### Plasma IPA measurement

The plasma of CAD patients was collected in a sodium citrate tube and promptly centrifuged at 12 000 rpm for 20 min at 4°C. Then the supernatant plasma was collected and stored at −80°C before analysis. Plasma IPA levels were quantified as previously described using ultra-performance LC–MS (UPLC-MS, Agilent) [31]. The mass spectrometer was operated in electrospray ionization (ESI) mode with optimized parameters. Quantification was performed using an external standard method with calibration curves ranging from 1 ng/mL to 250 ng/mL. Linearity was confirmed for all analyses.

### Platelet preparation, aggregation, secretion, P-selectin and activated integrin abundance, spreading, and clot retraction

The preparation and *in vitro* experiments of human and mouse platelets were described previously [56]. For platelet aggregation, 300 μL platelet-rich plasma (PRP) or 300 × 10^9^/L washed platelets were stimulated with agonists (ADP, thrombin, or collagen) under stirring conditions (1200 rpm) at 37°C. Platelet aggregation and ATP release were measured and recorded using a lumiaggregometer (Model 400 VS; Chrono-Log). To detect the release of P-selectin and the activation of integrin αⅡbβ3, resting platelets activated by thrombin (0.05 U/mL) for 5 min were incubated with PE-conjugated P-selectin and FITC-conjugated PAC-1 antibodies for 20 min. The levels of P-selectin expression and PAC-1 binding were subsequently analyzed using flow cytometry. For platelet spreading, platelets spreading on immobilized fibrinogen were stained with FITC-labeled phalloidin and then viewed using a Leica SPE confocal microscope. Platelet clot retraction was induced by thrombin (1.0 U/mL) in the presence of human platelet-poor plasma (PPP) and Ca^2+^ and recorded by taking photographs. The percentage of clot surface area and platelet spreading area was quantified using ImageJ software.

### Intravital microscopy of FeCl_3_-injured thrombosis in mouse mesenteric arteriole

Intravital microscopy of FeCl_3_-injured thrombus formation in mouse mesenteric arteriole was conducted as described previously with minor modifications [56, 57]. WT mice aged 6−8 weeks were randomly treated with vehicle, IPA (0.2 μg per mouse, intravenously (i.v.), single dose), clopidogrel (5 or 20 mg/kg, *per os* (p.o.), single dose), or clopidogrel (5 mg/kg, p.o., single dose) plus IPA (0.2 μg per mouse, i.v., single dose). Calcein-labeled platelets were then injected into the mice via the lateral tail vein at 5 min after IPA administration or 4 h after clopidogrel administration. Thrombosis was induced by 10% FeCl_3_ 5 min later. The time to the first thrombus (> 20 μm) formation and final occlusion was recorded using intravital microscope.

### Calcium measurement

Agonist-induced Ca^2+^ influx in platelets was conducted as described previously [58]. Washed human or mouse platelet suspension in Tyrode’s buffer without calcium was incubated with 5 μmol/L Fura-2 (Invitrogen) at 37°C for 30 min. After washing, different treatments were applied to the platelets at 37°C while they were continuously stirred for indicated time points. Fura-2 was excited alternately at 340 nm and 380 nm, and fluorescence emission was detected at 510 nm. Fluorescence signals were recorded using a fluorescence spectrophotometer (Duetta, HORIBA Scientific), and the values of 340 nm/380 nm ratio were converted into nanomolar concentrations of [Ca^2+^] by lysis with Triton X-100 and a surplus of EDTA according to the manufacturer’s instructions.

### Myocardial I/R model

Myocardial I/R model was induced by surgery as described previously [58]. Briefly, *ApoE*^*−/−*^ mice were anesthetized with 2% isoflurane gas and mechanically ventilated with a rodent respirator (inspiratory tidal volume being 250 μL at 130 breaths/min). A left thoracotomy was performed in the fourth intercostal space, followed by 45-min temporary LAD coronary artery ligation with 6.0 silk suture slipknot at its emergence site from the left atrium. Myocardial ischemia was confirmed by electrocardiographic changes of ST-segment elevation. The mice in the sham-operated group underwent the same procedure except for the ligation of the LAD.

### Echocardiography

Echocardiography was conducted 48 h after I/R injury using a Vevo 2100 instrument (Visual Sonics, Toronto, Ontario, Canada) with an MS-400 imaging transducer. Mice were anesthetized and placed supine. The chest was shaved, and the left parasternal short-axis view was recorded. Simultaneous transversal M-mode tracings were taken in the middle of the LV cavity. LV diameter at end diastole/systole (LVDd/s) and LV volumes at end diastole/systole (LVEDV and LVESV) were measured, and EF was calculated using Vevo 2100 software.

### Infarct area assessment

Mice were anesthetized 48 h after I/R injury, and the LAD artery was re-ligated at previous ligation. After injecting 1 mL 1% Evans blue dye (Sigma-Aldrich, St Louis, MO) into the LV cavity, the heart was immediately excised, rinsed, frozen, and sliced. Slices incubated in 1% TTC (Sigma-Aldrich) solution at 37°C for 20 min were then fixed in 4% paraformaldehyde and photographed. The blue area indicates the non-infarct area, while the unstained area represents the AAR. The red area shows viable myocardium and the white area indicates an infarct area. LV area, AAR, and infarct area were measured and calculated using ImageJ software.

### Immunohistochemistry

The mouse hearts were harvested after 7 days of reperfusion, and then fixed in 4% formalin, dehydrated in 70% ethyl alcohol, and made into paraffin sections. The slides were incubated with rabbit anti-CD62P antibody (1:50, Abcam) overnight at 4°C to stain platelets, and then with biotin-conjugated anti-rabbit IgG, avidin-linked enzyme peroxidase complex, and 4',6-diamidino-2-phenylindole (DAPI) as substrate at room temperature for 2 h. The stained slides were counterstained with hematoxylin, dehydrated, and photographed with an Olympus microscope. The positive stain in each section was quantified using ImageJ software.

### Western blotting

Washed platelet aggregation was induced by collagen under stirring conditions (1200 rpm, 37°C), and terminated 5 min later by 5× lysis buffer (50 mmol/L Tris, 10 mmol/L MgCl_2_, 150 mmol/L NaCl, 1 mmol/L NaF, and 1% NP-40, pH 7.4) containing protease inhibitor and phosphatase inhibitor. The platelet lysate was boiled with 6× loading buffer at 100°C for 5 min. Proteins were separated by SDS-PAGE, transferred to polyvinylidene fluoride membranes, incubated with antibodies, and then visualized with Tanon 2500muti (Tanon Science, Shanghai, China). The antibodies utilized in the study are listed in Supplementary Material.

### Statistical analysis

Unless otherwise stated, data were expressed as mean ± SEM. Data normality was determined by the Shapiro–Wilk test. Differences between two groups were analyzed by unpaired Student’s *t*-test. One-way ANOVA followed by Tukey’s multiple comparisons test for independent data was used when comparing > 2 groups. Two-way ANOVA followed by Tukey’s multiple comparisons test was used when > 2 groups and variables were compared. *P* value < 0.05 was considered to be statistically significant using Prism 8.0 (GraphPad Inc., San Diego, CA, USA).

## Supplementary Material

loaf002_suppl_Supplementary_Material

## Data Availability

The authors confirm that all the data supporting the findings of this study are available within the supplementary material and corresponding authors.
