# Supplementary material for: Intermittent fasting inhibits platelet activation and thrombosis through the intestinal metabolite indole-3-propionate
Source: Life Metab. 2025 Jan 29;4(2):loaf002. doi: 10.1093/lifemeta/loaf002 (PMC11897983; doi:10.1093/lifemeta/loaf002)
Supplement: loaf002_suppl_Supplementary_Material [file loaf002_suppl_supplementary_material.docx]

**
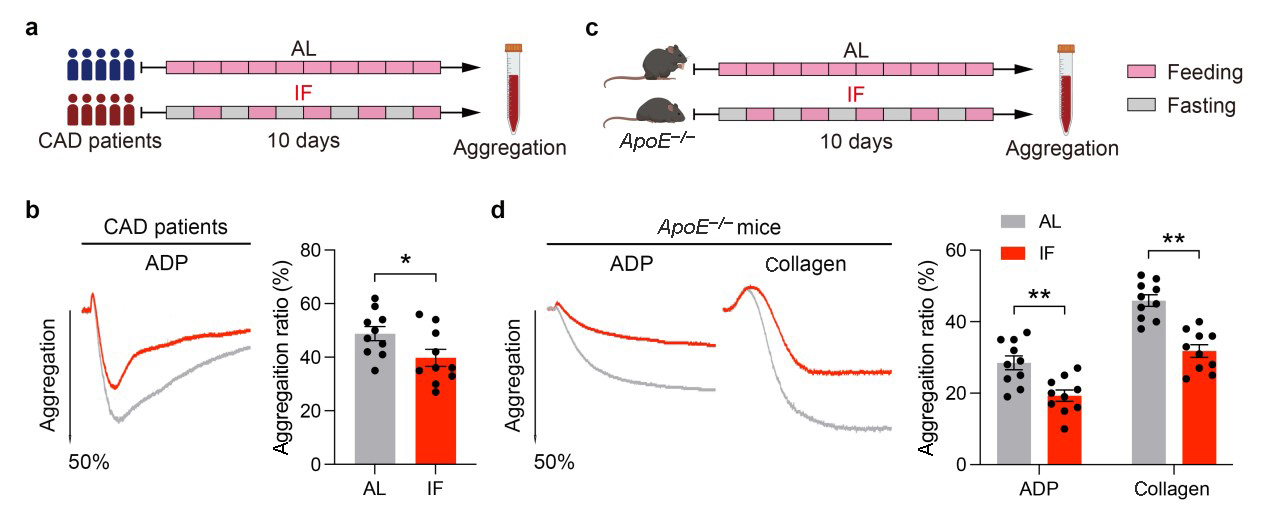
**

**Supplementary Figure S1** IF attenuates platelet activation, eliminating the effect of acute fasting. (a) Schematic diagram of AL or IF-diet treatment for CAD patients. CAD patients underwent 10 days of IF or AL diet and their platelets were collected immediately after the last eating day. (b) Representative tracings and summary results of human platelet aggregation induced by 10 μmol/L ADP, using platelet-rich plasma (PRP) from patients in (a) (*n* = 10, biologically independent individuals per group). (c) Schematic diagram of AL or IF-diet treatment for *ApoE^−/−^* mice. *ApoE^−/−^* mice were treated with 10-day IF or AL diet and their platelets were collected immediately after the last eating day. (d) Representative tracings and summary results of mouse platelet aggregation induced by 10 μmol/L ADP or 0.5 μg/mL collagen, using PRP from *ApoE^−/−^* mice in (c) (*n* = 10, biologically independent animals per group). Data are shown as mean ± SEM. ^*^*P* < 0.05; ^**^*P* < 0.01. Data were analyzed using unpaired Student’s *t*-test (b and d).

**
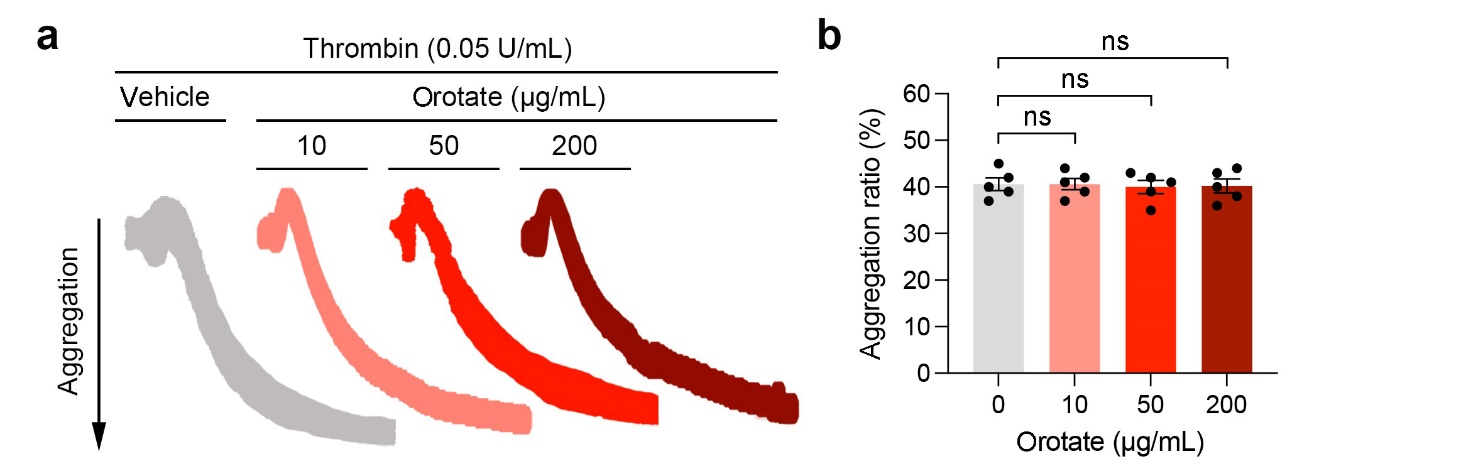
**

**Supplementary Figure S2** The effect of orotate on platelet aggregation at different doses. (a) Representative tracings of platelet aggregation induced by thrombin (0.05 U/mL). Washed human platelets were incubated with 10, 50 or 200 μg/mL orotate or vehicle for 20 min and then treated with thrombin. (b) Summary results of platelet aggregation ratio in (a) (*n* = 5, biologically independent individuals per group). Data are shown as mean ± SEM. ns, no significance. Data were analyzed using one-way ANOVA followed by Tukey’s multiple comparisons test (b).

**
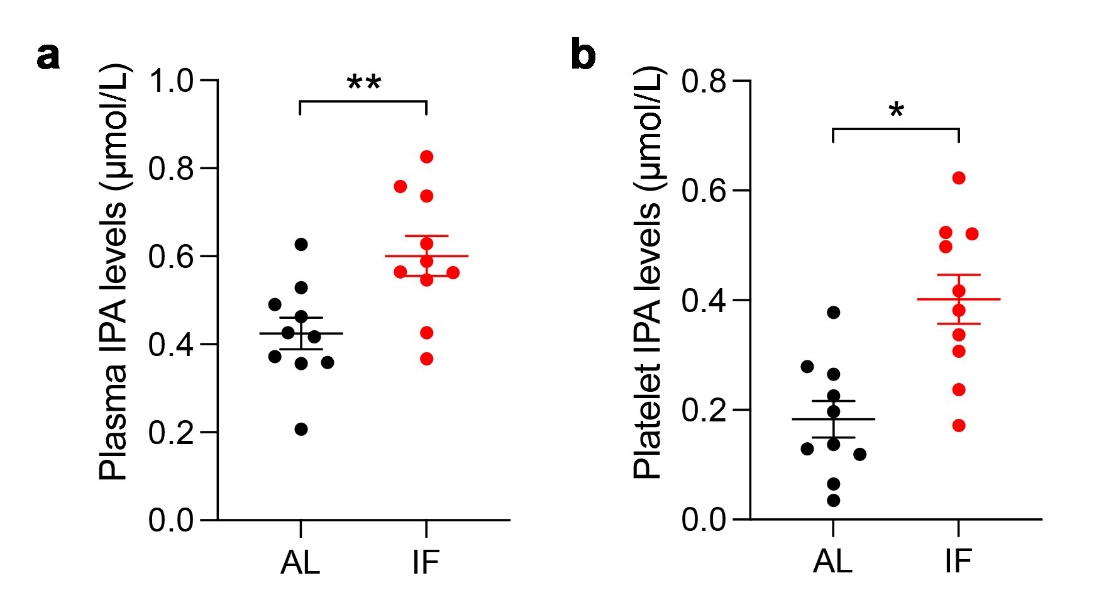
**

**Supplementary Figure S3** The IPA levels in both plasma and platelets from IF-diet human subjects are higher than those from AL-diet ones. (a) The levels of IPA in plasma of human subjects detected by LC-MS. (b) The levels of IPA in platelet lysis of human subjects. Data are shown as mean ± SEM. ^*^*P* < 0.05; ^**^*P* < 0.01. Data were analyzed using unpaired Student’s *t*-test (a and b).

**
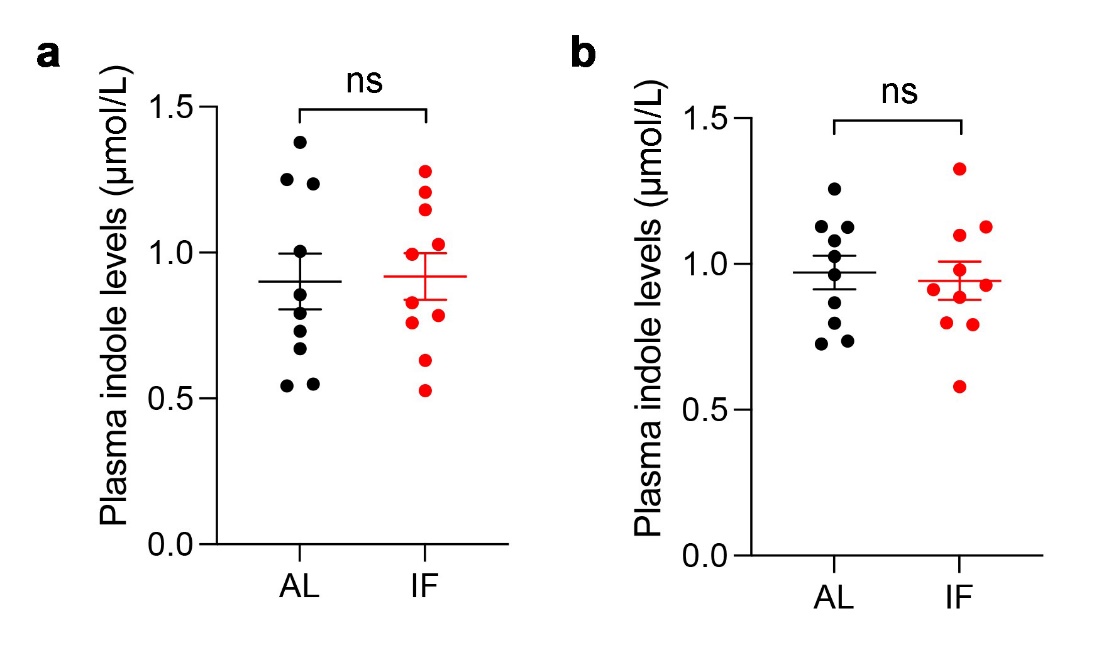
**

**Supplementary Figure S4** The indole levels in plasma of human subjects and mice undergoing AL or IF diet. (a) The plasma indole levels of CAD patients undergoing AL and IF diets. (b) The plasma indole levels of *ApoE^−/−^* mice undergoing AL and IF diets. Data are shown as mean ± SEM. ns, no significance. Data were analyzed using unpaired Student’s *t*-test (a and b).

**
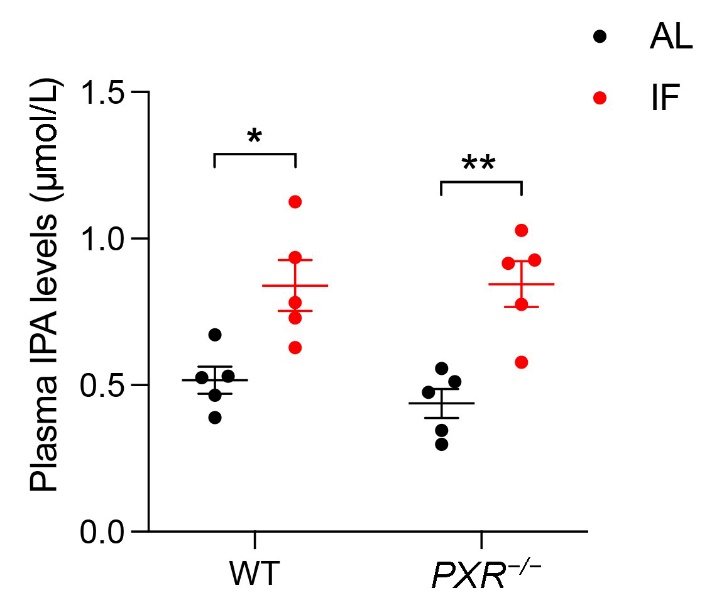
**

**Supplementary Figure S5** The IPA levels of IF-diet mice are significantly higher in both WT and *PXR^−/−^* mice. Data are shown as mean ± SEM. ^*^*P* < 0.05; ^**^*P* < 0.01. Data were analyzed using unpaired Student’s *t*-test.

**
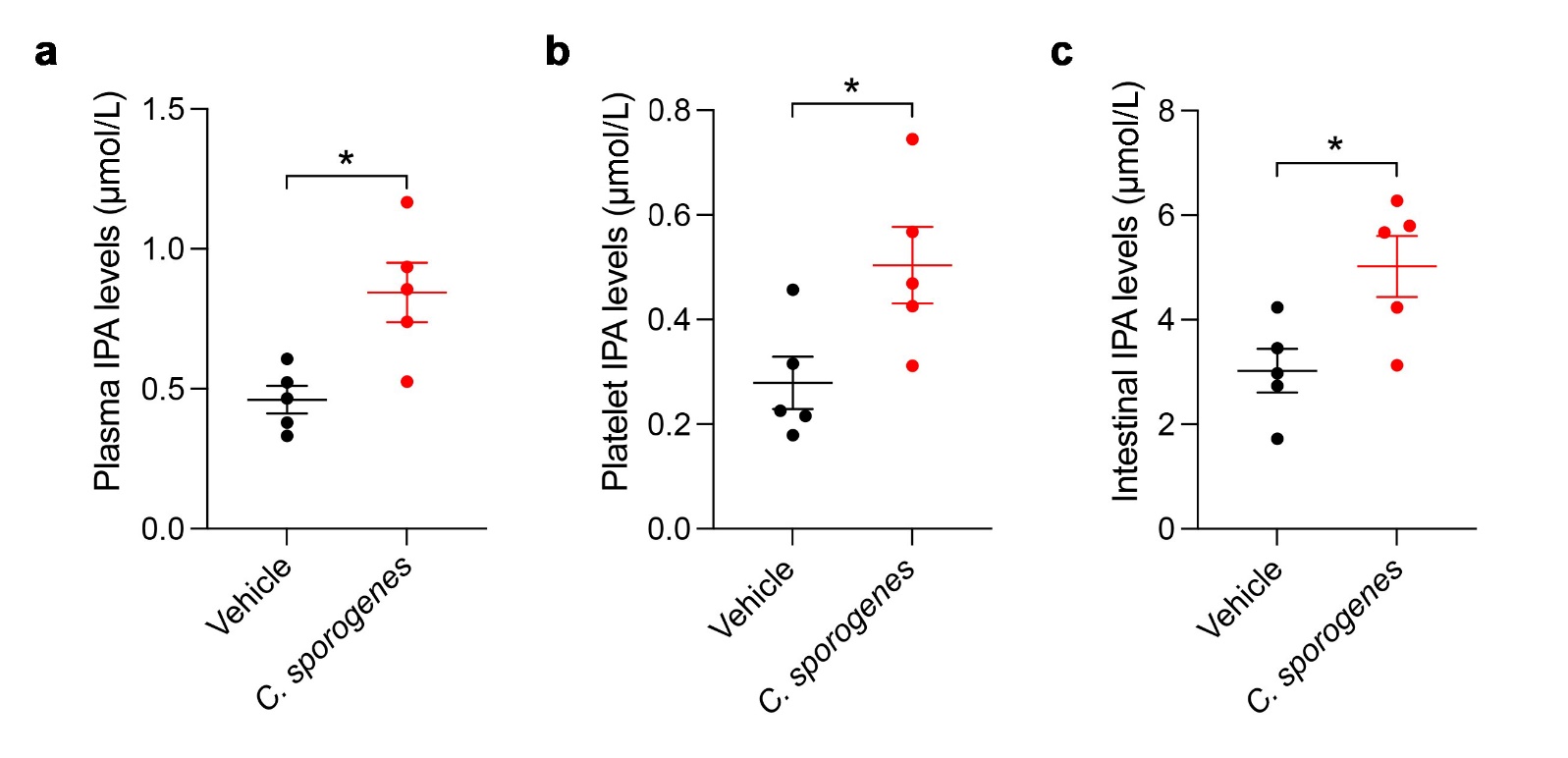
**

**Supplementary Figure S6** The IPA levels in plasma (a), platelets (b), and guts (c) of mice treated with *C. sporogenes*. Data are shown as mean ± SEM. ^*^*P* < 0.05. Data were analyzed using unpaired Student’s *t*-test (a, b, and c).

**
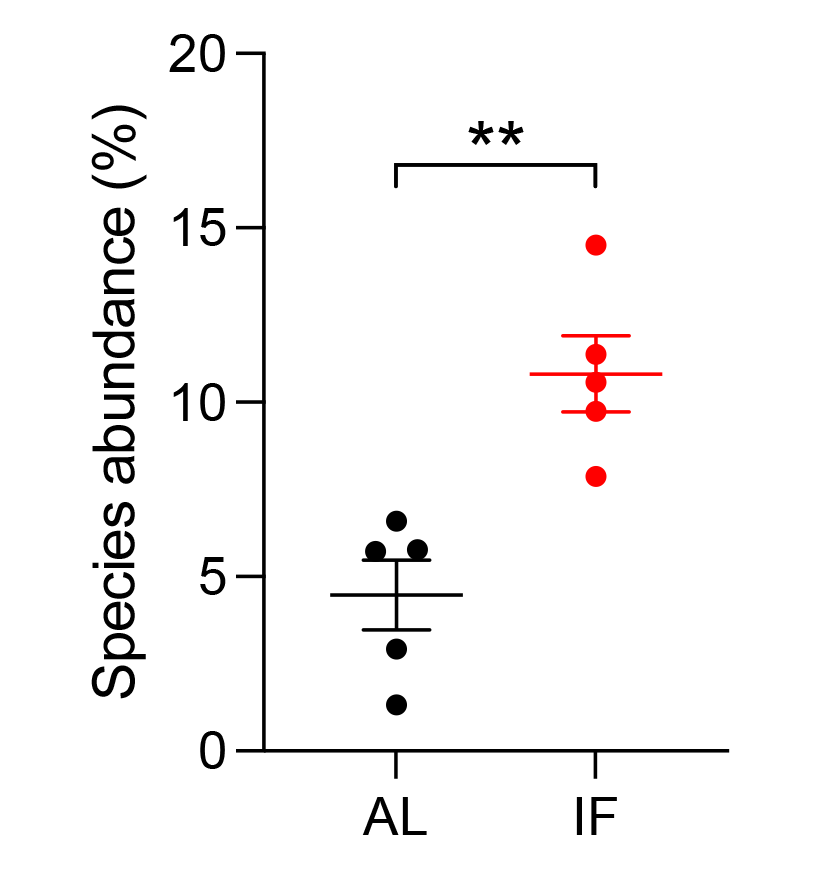
**

**Supplementary Figure S7** The abundance of intestinal *C. sporogenes* in the serum of mice treated with IF diet. Data are shown as mean ± SEM. ^**^*P* < 0.01. Data were analyzed using unpaired Student’s *t*-test.

**
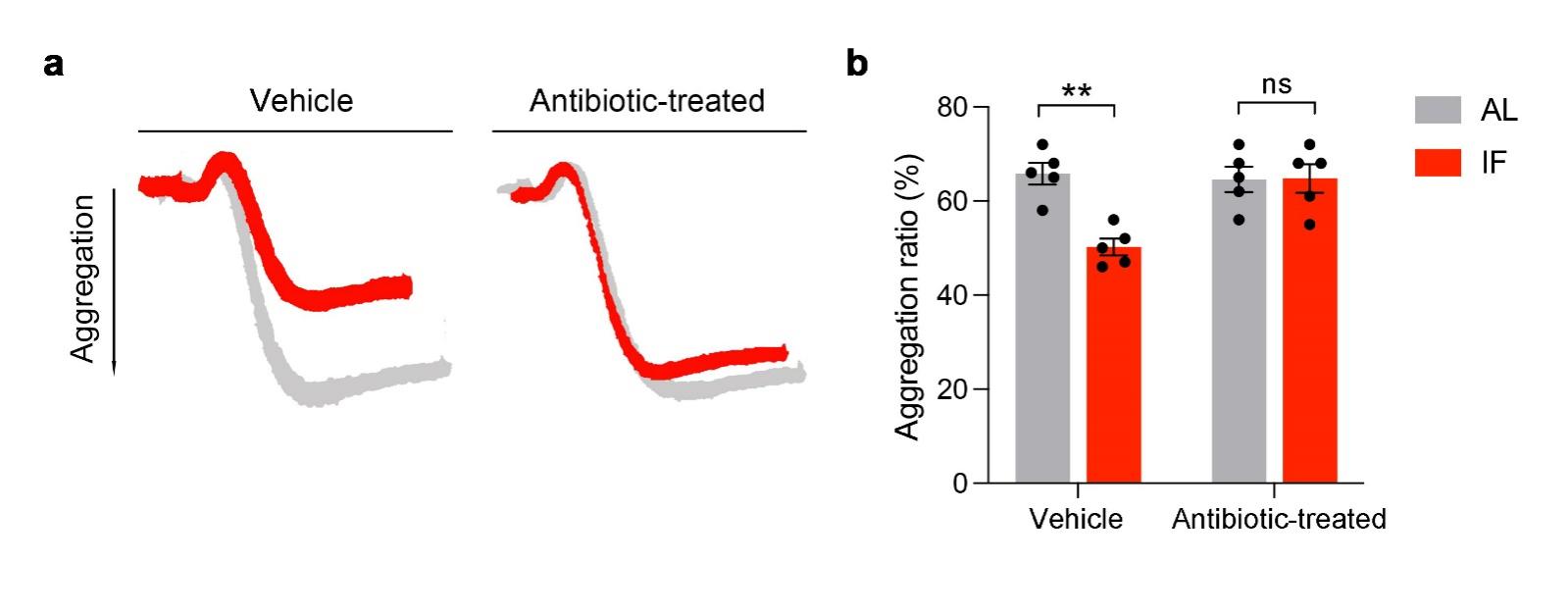
**

**Supplementary Figure S8** Antibiotic eliminates the inhibition effect of IF on platelet aggregation. (a and d) The representative tracings (a) and the summary data (b) of aggregation induced by 1.0 μg/mL collagen. Data are shown as mean ± SEM. ns, no significance; ^**^*P* < 0.01. Data were analyzed using unpaired Student’s *t*-test (b).

| Characteristics | Overall |
| --- | --- |
| *n* | 160 |
| Age (year) | 62.56 ± 10.72 |
| Male | 82 (51.2) |
| TC (mmol/L) | 4.13 [3.61, 4.61] |
| TG (mmol/L) | 1.38 [1.05, 2.27] |
| LDL-C (mmol/L) | 2.17 ± 0.79 |
| Platelet (×10^9^/L) | 199.00 [160.75, 238.00] |
| Hypertension | 60 (37.5) |
| Diabetes | 37 (23.1) |
| Statin | 29 (18.1) |

**Supplementary Table S1** Characteristics of CAD patients enrolled in the study. The results were presented as mean ± SD for normal continuous variables, median [IQR] for non-normal continuous variables, and *n* (%) for categorical variables. TC, total cholesterol; TG, triglyceride; LDL-C, low-density lipoprotein cholesterol.

| Antibody | Company (Catalogue No.) | Assay |
| --- | --- | --- |
| PE anti-human integrin αⅡbβ3 (PAC-1) | Invitrogen (MA5-28564) | flow cytomety |
| APC anti-human CD62P (P-selectin) | BD Biosciences (550888) | flow cytomety |
| PE anti-mouse integrin αⅡbβ3 (JON/A) | Emfret (M023-2) | flow cytomety |
| APC anti-mouse/rat CD62P (P-selectin) | Biolegend (148304) | flow cytomety |
| anti-phospho-Src (Y418) | Abcam (ab40660) | western blotting |
| anti-phospho-Lyn (Y397) | Abcam (ab300118) | western blotting |
| anti-phospho-Syk (Y525/526) | Abcam (ab58575) | western blotting |
| anti-phospho-LAT (Y200) | Abcam (ab68139) | western blotting |
| anti-phospho-PLCγ2 (Y1217) | Cell Signaling Technology (3871) | western blotting |
| anti-phospho-PKC | Cell Signaling Technology (2261) | western blotting |
| anti-GAPDH | Proteintech (60004-1) | western blotting |

**Supplementary Table S2** The antibodies used in the study.
